# Fighting fair: community perspectives on the fairness of performance enhancement in esports

**DOI:** 10.3389/fspor.2024.1330755

**Published:** 2024-03-12

**Authors:** Maximilian A. Friehs, Madison Klarkowski, Julian Frommel, Cody Phillips, Regan L. Mandryk

**Affiliations:** ^1^ Psychology of Conflict Risk and Safety, University of Twente, Enschede, Netherlands; ^2^ School of Psychology, University College Dublin, Dublin, Ireland; ^3^ Max-Planck Institute for Human Cognitive and Brain Sciences, Leipzig, Germany; ^4^ Human-Computer Interaction Lab, Department of Computer Science, University of Saskatchewan, Leipzig, Germany; ^5^ Interaction Media Group, Information and Computing Sciences, Utrecht University, Utrecht, Netherlands; ^6^ Faculty of Computer Science, University of Victoria, Victoria, BC, Canada

**Keywords:** esports, performance enhancement, fairness, doping, games, culture

## Abstract

**Aims:**

This study aims to explore community perspectives on enhancer usage in competitive gaming and esports, focusing on the perception of fairness and concerns about various potential performance enhancers.

**Methods:**

We conducted both qualitative and quantitative surveys to understand the competitive gaming community's opinions on different types of performance enhancers and their potential impact on esports. A thematic analysis was performed to identify key themes in how players rationalize their opinions.

**Conclusions:**

The gaming community differentiates between potential performance enhancers based on how problematic they are for the esports scene, with the most concern surrounding hard drugs, pharmaceuticals, and brain stimulation interventions. Participants who are more invested in competitive gaming tend to be more sceptical of enhancers and express greater concerns. Four themes were identified in the thematic analysis: (1) risk, (2) morality, (3) enhancer effects, and (4) regulation. To increase acceptance and perceived legitimacy in decision-making, it is recommended that regulators engage a variety of stakeholders in transparent decision-making processes when forming tournament rules and regulations. This will help address the fragmented regulatory landscape and prevent potential differences in the perception of tournament winners based on the governing body supervising the competition.

## Highlights

• The esports community has fairness concerns around the use of drugs, pharmaceuticals, and brain stimulation interventions.
• Enhancers that are encountered in everyday life (e.g., caffeine) are of limited concern.
• Esports regulators should engage in transparent decision-making processes when forming rules and regulation

## 1 Introduction

Our sense of “fairness” pervades every facet of society—we all hope to be treated fairly. A tension arises in that fairness is intrinsically subjective; perceptions of what is and isn't fair take into account a variety of circumstances, contexts, personal convictions, and cultural norms. This can give rise to conflicts in ideals. Discussions around fairness are typically predicated upon an individual or group having a perceived unfair advantage. Further, whether or not someone perceives an action as fair influences their willingness to take that action. This can have meaningful consequences, both in terms of out-competing vulnerable groups, and engaging in potentially harmful behaviours in order to confer an advantage. As such, understanding community perspectives around fairness is critically important for maintaining community standards and establishing effective regulations to keep people safe. Fairness is of special importance in the context of competition, such as in sports. The protection of fairness in professional sports has led to the creation of institutions such as the World Anti-Doping Agency (WADA), whose mission statement expresses that they seek to “protect the spirit of the sport”—and that “drug-enhanced performance is incompatible with athletic (and human) excellence.” The WADA argues that drug regulation is necessary because athletes, in pursuit of achieving optimal performance, are generally more accepting of occupational and medical risk; and, as such, are willing to embrace novel methods of performance enhancement (1–3). Further, the narrow focus on maximising performance can lead to situations in which other individuals are harmed to pursue a specific goal (4).

There are many parallels between traditional sports and esports in terms of fostering a willingness to engage with novel methods of performance enhancement. Popular esports such as League of Legends have between 10 and 11 million daily active users, with over 150 million registered accounts. For professional players to stand out, they are expected to consistently perform at an exceptional standard. Performing at this standard is extremely difficult to achieve and maintain, as the competition is fierce and the stakes are high. In addition to large sponsorship deals, there are increasingly large prize pools for many esports tournaments—for example, in 2021, The International (the biggest Dota 2 tournament) had a prize pool of $40M (5). Outside of the competitive pressure of the tournament context, there is also pressure to perform from many sources, such as team owners, coaches, sponsors, stream viewers, fans, and the esports community at large. With such high stakes, this performance pressure creates an environment in which players may be motivated to embrace novel methods of performance enhancement.

Among the esports community, there is a perception that players are willing to use or consume substances that may enhance performance (6)—especially caffeine and pharmaceutical stimulants like Adderall^TM^. This perception has been reinforced by examples of professional players that have publicly disclosed stimulant use for performance enhancement in tournaments (7), as well as professional players who have more generally claimed widespread consumption of stimulants at the professional level (8). Many esports tournaments are also sponsored by energy drink companies, where advertisements imply that there are performance-enhancing effects from consuming their products. While there are many medical risks associated with the use of stimulants as performance-enhancing drugs (9–11), the presence of performance-enhancing drugs in the esports context also raises questions around fairness and integrity. If some players are using performance-enhancing drugs while others are not, it may create an inequitable and uneven playing ground that undermines the spirit of fair competition. The esports community's perception of fairness will likely play a major role in determining the severity and extent of performance enhancement regulation within the industry.

While drug regulation and performance enhancement in primarily physical sports (e.g., tennis, cycling or weightlifting) have been the target of discussion, debate, and academic inquiry for decades (12–14), the problem remains novel in the context of esports. This problem is compounded by the fact that only a few studies exist that systematically investigate these types of performance enhancement in esports. As the popularity, perceived legitimacy, and financial investment into esports rises, so does the communal need to understand what is fair—and at what point a practice becomes unfair—in esports play. Some practices, such as installing third-party software to enhance in-game performance (e.g., aim assistance in first-person shooters), are generally considered unfair in competitive contexts (15). However, other cases are less clear cut—especially in regards to food supplements, drugs, pharmaceuticals, and even non-invasive brain stimulation technologies. It should be noted that there is a semantically important difference between enhancement and doping: While enhancement is a general term referring to any method by which performance can be increased, doping is more specific in that it commonly refers to the use of forbidden substances (e.g., pharmaceuticals) to maximize performance. Thus while for example the use of mental training, food supplements and biofeedback may all be performance enhancement, the use of anabolic steroids may be considered doping if forbidden by the sport association.

Due to the absence of universal and consistent rules and regulations in esports, a lack of industry-wide oversight, and continued discussion and scandals surrounding professional player intake of performance-enhancing substances (16, 17), there is a clear and urgent need to understand perceptions of performance enhancement in the gaming community. Acquiring this knowledge will allow developers and esports regulatory bodies to make informed decisions commensurate with community expectation and perception, and generate insights about esports players’ and spectators’ own relationships with performance enhancements in digital competitive contexts (18).

### 1.1 Scope of the paper

To support esports regulation, we need an in-depth understanding of community perspectives on performance enhancement in esports. In this paper, we examine how gamers of varying professionalization levels rationalize their opinions about the fairness, ethics, and regulation of enhancer usage in esports. We determined the extent to which gaming communities perceive enhancer usage as fair, and explore what kind of regulations community members propose based on their concerns about performance enhancers. We discuss whether or not current practices are sufficient from an ethical and regulatory perspective to appropriately deal with increasing enhancer usage. The fragmented regulatory landscape in esports may lead to a different perception of tournament winners based on which governing body supervised the competition, and this in turn may affect the perceived legitimacy of the sport as a whole. To guide more specific recommendations, we also examined whether or not perception differs based on the background of the players (e.g., esports athletes vs. casual players and viewers), as well as the type of enhancer.

To guide our investigation, we defined and answered the following set of research questions:

• RQ1: How do game communities perceive fairness of performance enhancers in esports?
  ○ RQ1a: How fair do players consider performance enhancers in esports? Does this judgement depend on the enhancer?
  ○ RQ1b: How concerned are players about the current state of ethics and regulation of performance enhancers in esports? Does this judgement depend on the enhancer?
  ○ RQ1c: Are there differences in perceived concerns and fairness between different types of players?
• RQ2: How do players rationalize their opinions about fairness, ethics, and regulations of performance enhancers in esports?

To answer these questions, we conducted a mixed-methods study. We presented several reddit communities with a survey asking a combination of open and closed questions. We leverage the innate pseudonymity of reddit’s forums, affording users a sense of privacy, and the online disinhibition effect (19), ultimately hoping to gather honest responses that are not affected by social desirability biases (20). The primary contribution of this work is an overview of community sentiment surrounding the fairness of performance enhancement in esports. Further, recommendations for esports organizations and regulators are discussed.

## 2 Background

Our research is grounded in prior work on regulations, justice, and legitimacy. In this section, we discuss regulations against cheating, as well as the challenges with enforcing regulations; models and facets of justice; and legitimacy and its relations to injustice.

### 2.1 Enhancer effects and regulatory challenges

Regulations in traditional sports are largely based on the need to maintain competition fairness. However, in some situations the perception of fairness is inherently subjective. In a game itself rules are ideally set out clearly from the start and as long as everybody adheres to those rules, the game would be considered fair. However, regular discussions about the interpretation or the fairness of rules emerge, highlighting the need for a referee. In sports involving physical contact such as judo or rugby some rules exist to ensure the bodily wellbeing of athletes. In esports the physical wellbeing of athletes typically is not a risk and altercations on the pitch less of a concern. In the context of regulating esports, it is critical to understand why people play esports—and, further, why should they play “fair"? Motivations for professional-level athletes (e.g., the pursuit of fame, fun, financial incentives) may not be related to the reason why a professional player of any (e)sport adheres to certain rules and regulations. Further, the impact of regulations may extend beyond the competition—affecting preparatory training and the private lives of athletes, as well as lifestyle choices (21, 22). Recently, amateur athletes have been in the focus of anti-doping studies and interventions. Amateur athletes lack the medical support team that can mitigate the risk of using certain drugs to enhance performance and a harm-reduction approach may be needed to dis-incentivize or at least reduce the negative health-related outcomes of enhancer usage (23–25) Moreover, regulation itself is often problematic: for example, by stating which specific drugs are prohibited, slightly modified substances may circumvent a ban. In turn, competitors may seek out the consumption of new—and potentially unsafe—drugs that have not yet been prohibited (12, 26). Further, opaque regulations allow for arbitrary judgements that may be influenced by personal biases (e.g., favouritism).For pharmaceuticals and other drugs, it is critically important to consider the varying effects between individuals. For example, the effect of one medication may differ depending on the underlying hormonal profile of a competitor—and so, sensible regulation must be guided by a strong scientific base, and regulatory bodies should consist of a diverse group of individuals (12, 27).

Finally, an additional complexity in regulation is that of medical exemption. Some athletes may require certain medication to deal with an underlying medical problem (e.g., the use of amphetamine salt compounds like Adderall to manage the symptoms of ADHD). However, evidence from Olympic sports shows that both approving and denying a medical use exemption has its issues such as the undertreatment of athletes with medical issues (e.g., ADHD) (28–30). Athletes with asthma are an intriguing example. It is perceived in multiple sports communities that the intake of asthma medication may enhance performance, which leads to a negative perception of athletes with a corresponding therapeutic use exemption for their inhaler (31). Consequently, athletes that may actually need an inhaler refuse therapy in order to avoid being stigmatized. Thus there is a urgent need to change the perception among the athletes with regards to asthma medication, to maintain respiratory health (32). It is possible that athletes may falsely report their symptoms to get access to medically unnecessary prescriptions, and an unjustified medical use exemption. This is especially easy in the context of telehealth—there are websites that are specifically designed to allow people to receive an ADHD diagnosis based on their answers to a standardized test, where scores to receive a diagnosis have been publicly posted online. On the other hand, if a legitimate medical exemption is denied for reasons such as favoritism, the accessibility and integrity of the sport come into question. An ethical tension arises in that allowing people with a legitimate medical use case to compete is important from an access and equity lens, yet this opens a loophole that may promote potentially harmful behavior in players willing to take medical risks.

Thus, it is difficult to create a perfect regulatory framework. Although the WADA has long been the key player in policing illegal performance enhancement, in 2020 the United States of America have singed the Rodchenkov-Act into law, which allows the USA to pursue organizations and athletes involved in doping even outside national borders and charge them with criminal offences (House Bill 835, Public Law No. 116-206). Essentially, with this new self-empowerment the USA is grabbing power from the WADA and creates a second anti-doping policy leader, but the specific consequences of this action remain to be observed (33). Regardless of who holds the regulatory power, regulators need to rely, at least in part, on athletes’ own moral conduct. However, previous research has demonstrated that at least 10% of surveyed athletes in traditional sports admit to cheating via doping (13, 34); when considering the potential influence of social desirability bias on self-report responses, the actual usage rate may be even higher. Recently Gleaves and colleagues reviewed literature from 1975 to 2021 about the prevalence of doping in competitive sports (35). When considering only, in their view, high-quality studies 50% report below 5% prevalence rates, 30% between 5%–20% and 20% of studies 30% or higher prevalence ratings. Motivations for cheating are often driven by financial and fame-oriented desires: for example, one study revealed that the temptation to cheat is predicted by a lack of self-control, high impulsivity, and a desire to get rich, as well as social and moral values of the individual (36). A desire for financial gain appears directly predictive of cheating behavior—especially in private situations where the individual thinks they are not supervised. Similarly, Charness and colleagues showed that cheating behavior in the absence of financial incentives was reduced (37). These results are in line with a recent meta-analysis on the topic (18).

### 2.2 Models and facets of justice

When trying to resolve ethical tensions, it is important to consider the concept of justice. The perception of justice is inherently subjective and often moderated by the role of the stakeholders involved. As such, the concept of justice is multidimensional and subject to a variety of perspectives (38, 39). In the context of fairness in esports, there are four important aspects to consider: first, distributive justice describes the fair allocation of resources; second, procedural justice describes the procedures by which a resource is distributed; third, interpersonal justice refers to an individual's (e.g., an employer or judge) perception of fair treatment of another person (e.g., an employee or defendant); and fourth, informational justice describes clear, transparent, and needs-oriented communication. For example, in a competition format, a specific rule might not be communicated clearly to all teams (informational injustice), or an official may be more lenient towards one team compared to another (interpersonal injustice). Consequently, the process of determining the winner of a competition may be called into question (procedural injustice), and it may be perceived that a winning team won undeservedly (distributive injustice).

### 2.3 Legitimacy and its relation to (in)justice

According to Tyler's Legitimacy Theory, justice and injustice are tied to the perception of legitimacy (40): the belief that an authority, and its power, is justified. In the context of competition and fairness, a governing body that is perceived to be legitimate is crucial for the adherence to rules and the acceptance of regulatory actions. Research shows that procedural and distributive justice are good predictors of legitimacy (41–44). This connection between perceived fairness and legitimacy is in line with studies of athletes evidencing a desire for the fair, equal, and transparent testing of illicit substances (45). If judges and other officials do not adhere to established procedures, competitors may feel betrayed, which in turn may reduce motivation and future rule adherence. However, people frequently do not have all the information necessary to make a truly objective statement about whether or not an outcome or decision is fair or unfair. Thus, there certainly is a degree of uncertainty involved in most judgements. The Uncertainty Management Theory posits that when information about how to assess fairness is incomplete, individuals will turn to judgements of fairness on other dimensions (46–48). Practically speaking, individuals will use procedural justice judgements to evaluate the fairness of the outcome. A process is perceived as just if all individuals can expect an outcome proportional to their inputs, and that relation is identical across all individuals. Consequently, if an institution can guarantee such subjectively fair procedures, it may be more likely to be perceived as legitimate and morally credible (49).

## 3 Methods

To answer our primary research questions (RQ1. How do game communities perceive fairness of performance enhancers in esports?; RQ2. How do players rationalize their opinions about fairness, ethics, and regulations of performance enhancers in esports?), we conducted an online survey investigating perceptions of enhancers in esports. This section details our data collection approach, survey instruments, exclusion criteria, and analysis methods.

### 3.1 Data collection

The survey was advertised on the social news aggregation website reddit, on selected “subreddits” (that is, subforums that cater to particular topics). Moderator approval was acquired prior to advertising and posting on a subreddit, with non-permitted subreddits removed from the pool. In total, the survey was advertised on 27 subreddits between the 1st and 15th of December, 2021. For a complete list of subreddits used, please refer to the Supplementary Material (see https://osf.io/65qzp/). Compensation was offered in the form of an opt-in raffle for one of five $100USD Amazon gift cards. Overall, 664 participants completed the survey. The study has ethical approval from the University of Saskatchewan Ethics Board.

### 3.2 Instruments

To assess the overall perception of enhancers in esports and participant opinion on the topics of fairness, regulation, and enhancement usage, we employed a combination of closed-ended and open-ended items. Participants provided demographic information (e.g., age and gender), their preferred game genres, and their self-identified “gamer type”. The six different gamer types included: (1) full-time professional esports athlete (i.e., esports related earnings make up most of your income), (2) part-time professional esports athlete (i.e., a portion of your income is esports related), (3) amateur esports athlete (i.e., you play in an organized team but earn little to no money with esports), (4) competitive gamer (i.e., you play competitive games regularly), (5) casual gamer (i.e., you do little to no competitive gaming), and (6) speedrunner (e.g., a competitive player that plays the with the intent of completing it as quickly as possible given a certain ruleset). The researchers chose these categories.

For the purpose of the survey, we established five discrete categories of performance enhancers: (1) Food & Food Supplements (e.g., caffeine, Tyrosine, sugar), (2) Pharmaceuticals (e.g., Modafinil, painkillers, benzodiazepines), (3) Drugs that are Commonly Socially Accepted (e.g., alcohol, nicotine, cannabis), (4) Drugs that are Commonly Socially Not Accepted (e.g., psychedelics, opioids), and (5) Non-Invasive Brain Stimulation (e.g., transcranial direct current stimulation)^1^. Participants were provided with a brief description of and introduction to each enhancer category, with examples included for each. Prior to deployment, the survey was piloted internally to ensure ease of understanding and clarity. The survey comprised of five blocks—the first four of which focused on questions concerning the aforementioned performance enhancer categories, and the final block contained open-ended questions concerning fairness, ethics, and regulation^2^. We acknowledge that the classification system presented here is not the sole method for categorizing performance enhancement methods. An alternative system, for instance, might classify performance-enhancing drugs into categories such as stimulants, depressants, cannabinoids, hallucinogens, hypnotics, and dissociatives. However, classifications of this nature introduce unwarranted ambiguities, as seen in instances like both caffeine and methamphetamine falling under the umbrella of stimulants. Further, some of our classifiers were more technical compared to others; Non-Invasive Brain Stimulation for example is a technical term referring to a variety of methods of electrical or magnetic brain stimulation that directly affect nerve cell activity in the brain.

#### 3.2.1 Fairness

The fairness questions were adapted from the Distributive Justice Subscale by Colquitt and colleagues (38, 50). For each enhancer category, participants were asked to respond on a 5-point Likert scale (“definitely not” to “definitely yes”) to the question, “If somebody was using [specific performance enhancement category] and was winning a tournament, how would you perceive their success?”. The four items to be rated included, “Would the success be reflective of the effort put into the tournament?” (Effort), “Is the success appropriate for the work the player put in?” (Success), “Does the success reflect the individual contribution to the tournament?” (Contribution), and “Would the success be justified given the performance?” (Justified). Based on these items an overall scale score was calculated.

#### 3.2.2 Concerns

For each enhancer group, we asked questions about whether or not participants believed a certain subgroup of performance enhancers should be regulated, or if the use of an enhancer would constitute an unfair advantage. Participants were asked to indicate the degree of how concerned they were from 0 (‘definitely not’) to 100 (“definitely yes”). This block included four questions: “Would you have any ethical concerns?” (Ethics), “Do you think the usage of this enhancer should be regulated by official esports organizations?” (Regulation), “Would you consider the usage of this enhancer as a form of unfair advantage?” (Cheating), and “Do you think somebody winning in a competition under the influence of this enhancer should be disqualified?” (Disqualified). Based on these items an overall scale score was calculated.

#### 3.2.3 Open questions

After each segment of questions relating to fairness for each enhancer, participants could optionally reply to the following open-ended question: “Is there a context in which you believe the enhancer to be fair or unfair to use?” Further, toward the end of the questionnaire, participants were prompted to reply to the following open-ended questions:

• “Please share your thoughts about the regulatory implications of using different enhancers for gaming purposes in general. Do you think all or only certain enhancers should officially be regulated in esports tournaments?”
• “Please share your thoughts about the fairness or ethical implications of using different enhancers for gaming purposes in general. Would you consider enhancement as an unfair advantage or would that depend on the circumstances or the enhancer used?”
• “Where would you draw the line between fair and unfair advantages gained by the use of enhancers? Could you describe a context in which enhancements are fair use, and a context in which enhancements are unfair?”

### 3.3 Data collection and reduction

The survey was advertised on selected subreddits (that is, subforums) on the website reddit.com. Prior to advertising on a subreddit, the authors sought approval from subreddit moderators. In total, the survey was advertised on 27 subreddits (for a complete list, refer to the Supplementary Materials https://osf.io/65qzp/) between the 1st and 15th December 2021. All participants could opt into a raffle to win one of five $100 USD Amazon gift cards. Overall, 664 participants completed the questionnaire Suspected inauthentic data were excluded from further analysis based on the following criteria: (a) average time to completion was below 1.5 s per question, not including the optional open-ended questions, (b) implausible data entry, (c) duplicate replies, indicative of bot or script usage. Based on these criteria, 98 participants were removed from the sample.

### 3.4 Analysis procedure

#### 3.4.1 Quantitative questionnaire data

The analysis of the questionnaire data involved two phases. First, the quantitative questionnaire data (i.e., responses to the closed questions) were analyzed in order to characterize the sample and find differences between different self-reported gamer types with regard to their level of competitive professionalism. However, due to the unequal group sizes based on self-reported type (i.e., fewer professional esports athletes compared to casual players) complicating an interpretation of inferential statistical data, we also performed a cluster analysis, with the aim to distribute the sample into more homogeneous subgroups. A cluster analysis groups individual datapoints in such a way that data in the same group (i.e., a cluster) is more similar (based on certain input variables) to each other than to those in other clusters.

#### 3.4.2 Qualitative questionnaire data

The open questions asked about the perceptions of fairness specific to enhancer categories, as well as the general perception of regulation and the ethical considerations surrounding enhancer usage. The general analysis procedure for the open questions was the same across questions and followed thematic analysis procedures (51, 52). In the first step, one author generated an initial codebook for the enhancer-specific question of fairness. Second, four of the authors (including the author who generated the initial codebook) each coded 25% of all non-blank replies for that question. Third, to reach a consensus, the authors engaged in discussion throughout the coding process to ensure commensurate understanding. Steps one to three were iterated upon until all authors agreed on the final themes, and their descriptions.

### 3.5 Positionality statement

All authors possess a background in games user research, and have undertaken previous scholarship in the context of online competitive gaming (and, specifically, the examination of performance enhancement in these spaces). Additionally, all authors possess prior experience playing popular esports titles (such as DotA 2, Counter-Strike, and Player Unknown's Battlegrounds). As such, the authors have examined and interpreted the findings described within this work through the lens of games academics and players, more broadly, and scholars of performance enhancers in games, more specifically.

## 4 Results

This section details the results of our online survey. To structure the results of our mixed-methods study, we report results in relation to our research questions.

### 4.1 Demographics

After data filtration, the dataset consisted of 566 individuals (mean age = 25.88, SD = 6.59). The majority of individuals identified as men (*n* = 477), whereas about 12% identified as women (*n* = 66). 11 individuals identified as non-binary, 8 preferred not to disclose their gender and 4 indicated the wish to self-describe the gender they identified with most (e.g., trans woman or genderfluid). Listed in order of frequency, participants indicated the following gamer identity: Competitive gamer (*n* = 325, 57.4%), casual gamer (*n* = 121, 21.4%), amateur athlete (*n* = 74, 13.1%), part-time professional player (*n* = 26, 4.6%), full-time professional player (*n* = 12 2.1%) and speedrunner (n = 8, 1.4%).

### 4.2 RQ1a: how fair do players consider performance enhancers in esports? Does this judgement depend on the enhancer?

The reliability of this scale for all enhancers was satisfactory (Cronbach's α = .90–.95). The overall fairness score was submitted to an ANOVA with enhancer type as the grouping variable and all F-values were Greenhouse-Geiser corrected due to the violation of the sphericity assumption. Results reveal a significant main effect of enhancer type (F(3.55, 2003.09) = 123.52, *p* < .001, *η*^2^ = .18). Descriptively, pharmaceuticals and not accepted drugs are perceived as least fair, followed by brain stimulation and accepted drugs. Food supplements however were perceived as relatively fair in comparison. For details, see Table 1.

**Table 1:** Facets of perceived fairness with regards to different enhancers.

	Food & food supplements	Brain stimulation	Drugs (accepted)	Drugs (not accepted)	Pharma-ceuticals	Question means
Effort	3.97 (1.21)	3.24 (1.21)	3.55 (1.39)	3.03 (1.48)	3.07 (1.30)	3.36 (.95)
Success	3.99 (1.20)	3.16 (1.21)	3.61 (1.37)	2.99 (1.49)	3.03 (1.31)	3.34 (.93)
Contribution	3.96 (1.21)	3.21 (1.17)	3.55 (1.37)	3.03 (1.47)	3.05 (1.31)	3.36 (.94)
Justified	3.98 (1.22)	3.12 (1.23)	3.66 (1.35)	2.97 (1.48)	2.98 (1.31)	3.34 (.95)
Grand average	3.98 (1.12)	3.18 (1.06)	3.59 (1.25)	3.01 (1.38)	3.03 (1.21)	

Participants could rate each enhancer on each facet of concern from 1 (unfair) to 5 (fair). Standard deviations in brackets. Note that the four components together make up the Grand Average, which is indicative of the overall scale value with regard to a specific enhancer.

**Figure 1:**
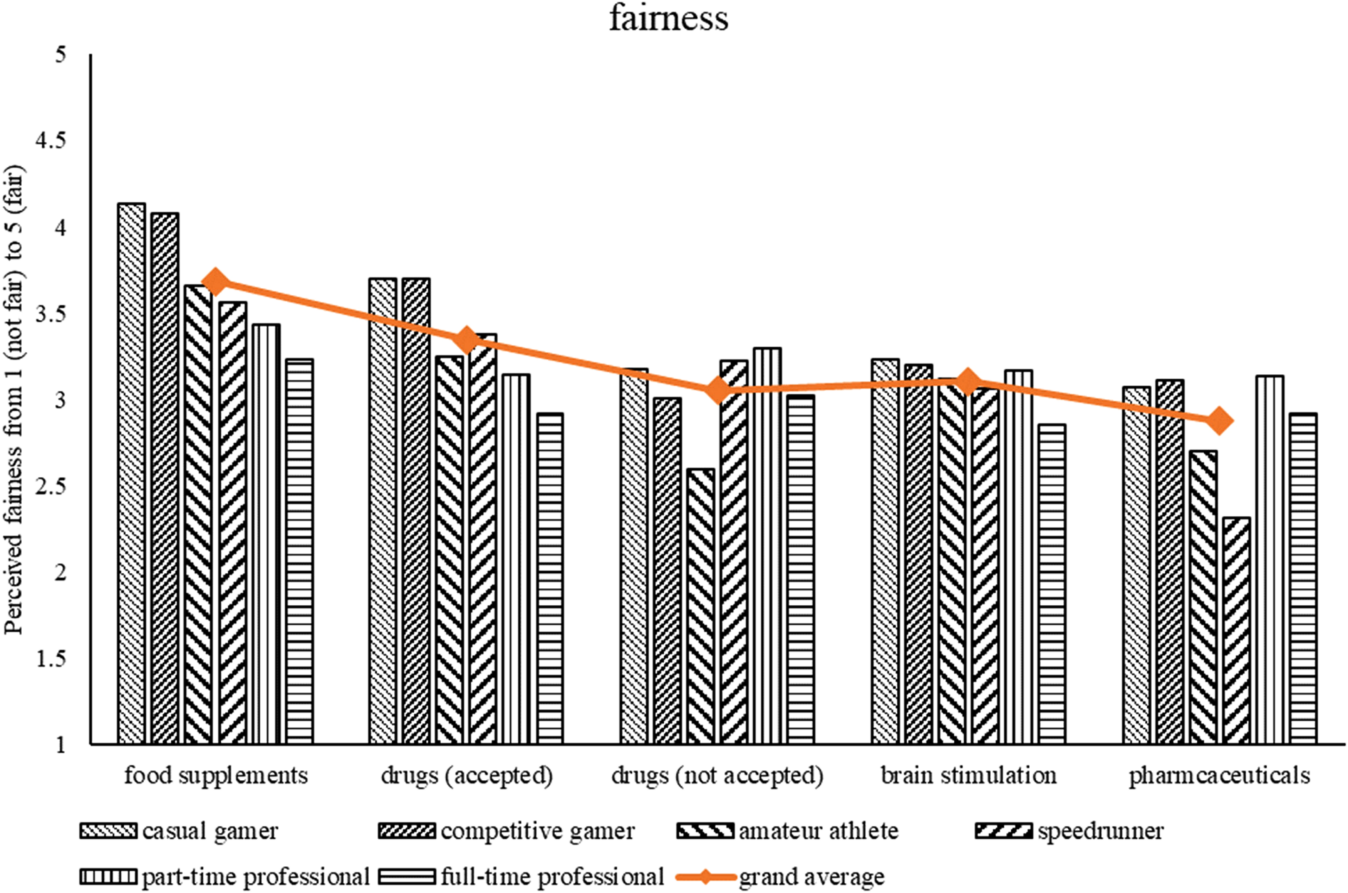
Perceived fairness as a function of enhancers and gamer type. Higher values indicate higher perceived fairness.

To further disentangle the enhancer-specific effects on fairness perception (see RQ1a), post-hoc analyses were performed using the overall fairness scores. Pairwise comparisons between each enhancer revealed that pharmaceuticals and not accepted drugs were perceived as least fair (*p* *< *.01 for all other enhancers) and did not significantly differ from each other (*p* = .65). Brain stimulation was perceived as slightly more fair compared to accepted drugs (*p* *< *.05) and food supplements were considered fairer than all other enhancers (*p* *< *.001). See Figure 1 for a visual representation of results.

### 4.3 RQ1b: how concerned are players about the current state of ethics and regulation of performance enhancers in esports? Does this judgement depend on the enhancer?

The reliability of this scale for all enhancers was satisfactory (Cronbach's α = .83–.90). To test for differences in concerns about enhancers, we performed a one-way ANOVA with enhancer type as the independent variable. Since the sphericity assumption was violated, all statistics were Greenhouse-Geiser corrected. Results reveal a significant main effect of enhancer type (F(3.54, 2000.07) = 257.84, *p* < .001, *η*^2^ = .31). Descriptively, participants are most concerned about pharmaceuticals, not accepted drugs, and brain stimulation, and least concerned about food supplements. For details see the descriptive statistics see Table 2.

**Table 2:** Facets of concern with regards to different enhancers.

	Food & food supplements	Brain stimulation	Drugs (accepted)	Drugs (not accepted)	Pharma-ceuticals	Question means
Ethics	23.00 (27.54)	53.92 (33.00)	38.64 (34.45)	67.21 (35.45)	56.22 (33.62)	47.80 (22.72)
Regulation	29.02 (31.75)	65.72 (32.10)	48.53 (36.24)	67.16 (36.19)	63.55 (33.23)	54.80 (23.47)
Cheating	31.04 (31.334)	59.76 (30.73)	33.76 (32.21)	44.15 (36.19)	57.85 (31.63)	45.31 (22.10)
Disqualified	22.48 (28.12)	56.62 (31.44)	36.77 (34.21)	59.29 (36.99)	51.64 (33.28)	45.36 (22.64)
Grand average	26.28 (26.08)	59.00 (26.52)	39.42 (29.41)	59.45 (29.30)	57.31 (28.39)	

Participants could rate each enhancer on each facet of concern from 0 (not concerned) to 100 (definitely concerned). Standard deviations in brackets. Note that the four components together make up the Grand Average, which is indicative of the overall scale value with regard to a specific enhancer.

**Figure 2:**
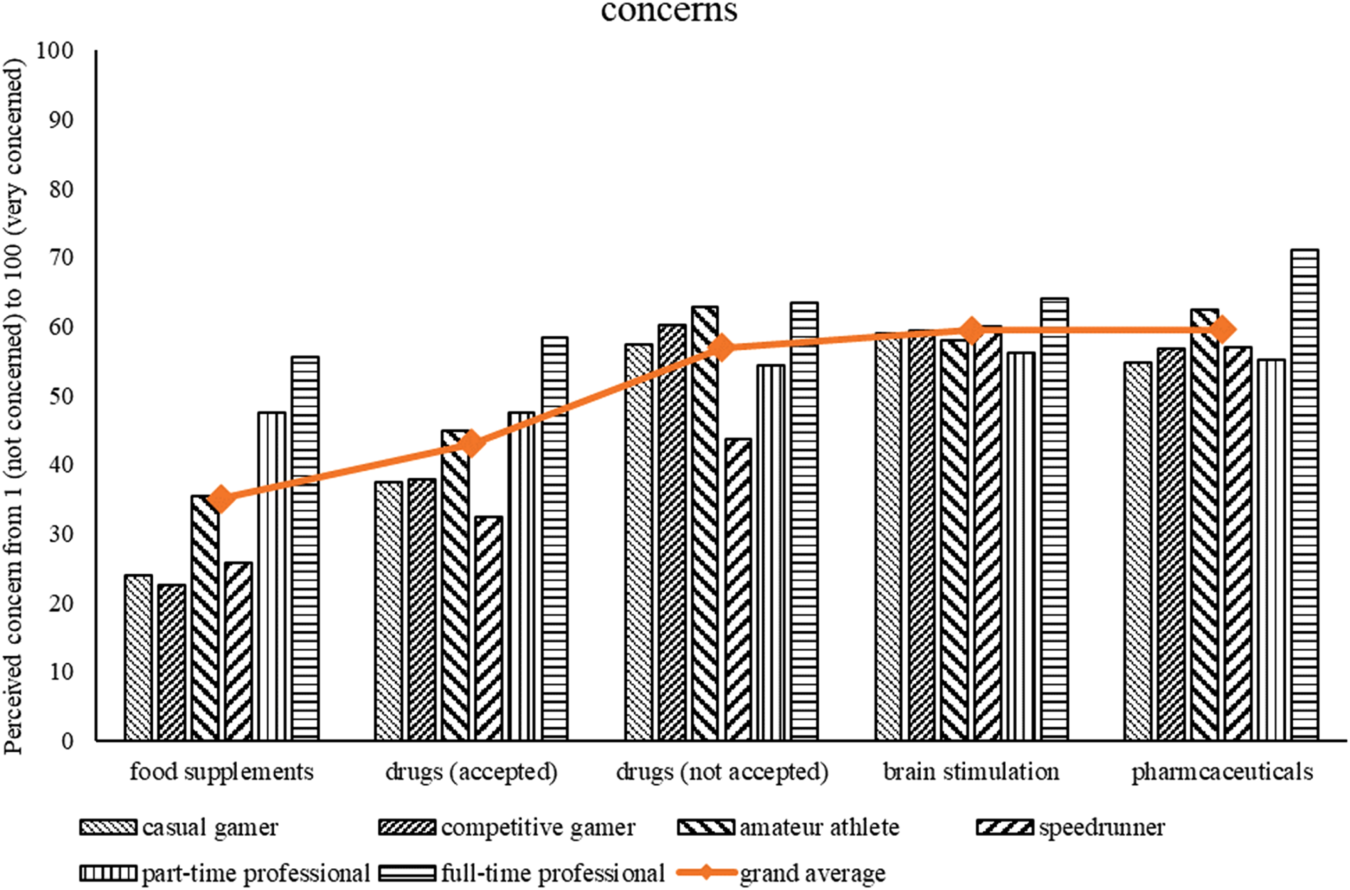
Perceived concerns as a function of enhancers and gamer type. Higher values indicate more perceived concern.

To further disentangle the results of RQ1b, post-hoc analyses were performed on the overall scale score. Pairwise comparisons between each enhancer revealed that pharmaceuticals, brain stimulation, and not accepted drugs were most concerning (all *p* < .001 compared to other enhancers) and did not differ from each other (*p*-values between.12 and.72). The concern for accepted drugs did differ from food supplements (*p* < .001). For a visual representation see Figure 2.

### 4.4 RQ1c: are there differences in perceived concerns and fairness between different types of players?

Based on the results from RQ1a and RQ1b (see Figures 1, 2) it seems that, descriptively, different groups of gamers (as indicated through the self-disclosed gamer identity) differ in perceived fairness and concerns about enhancers. In general, the more dedicated a player might be, the more concerned about enhancers they might be and the less fair they perceive them. Specifically, full-time professionals, amateur athletes, and speedrunners seem to be most concerned about enhancers—whereas casual and competitive gamers are less concerned. First, we correlated the scores from the subjective concerns and fairness questionnaires. Results reveal significant negative correlations of concerns for enhancers and fairness; brain stimulation (*r* = −.54, *p* < .001), accepted drugs (*r* = −.59, *p* < .001), not accepted drugs (*r* = −.62, *p* < .001), pharmaceuticals (*r* = −.58, *p* < .001), and food supplements (*r* = −.51, *p* < .001). This indicates that the higher the concern for a specific enhancer, the less fair an enhancer is perceived (and vice versa). Second, to further examine our interpretation, we conducted a k-means cluster analysis (2, 53)^3^. The goal is to create clusters that contain homogeneous data points, but are as heterogeneous as possible. Clustering allows the identification of participants that are similar regarding their perceived fairness of and concerns about enhancers. Variables were standardized before the analysis was carried out using the R-packages “cluster” (54) and “factoextra” (55). Based on several heuristics (i.e., best separation of measurement points, scree plot, silhouette method), the optimal number of clusters was determined to be two. For more details refer to the supplemental data (see https://osf.io/65qzp/).

Overall, 232 participants were in Cluster 1 (“tolerate”), characterized by low concern and high perceptions of fairness, and 334 participants in Cluster 2 (“troubled”), characterized by high concern and low perceptions of fairness. The clusters do somewhat correspond to the self-identified gamer identities. Notably, amateur athletes as well as part- and full-time professional players seem to overwhelmingly fall into the second cluster and perceive enhancers as more troubling. This is also true for competitive gamers, but to a lesser degree. Casual gamers as well as speedrunners are equally distributed between the two clusters. Note that the overall sample of speedrunners is low and thus, interpretation may be limited. For details see Table 3.

**Table 3:** Distribution of participants to one of the two clusters, as a function of their self-described gamer identity.

Gamer Identity	Cluster 1: tolerate	Cluster 2: troubled	*χ* ^2^	*p*
Casual gamers	61	62	0.01	0.93
Competitive gamers	142	183	5.17	.02*
Amateur athletes	18	56	19.51	.00000999***
Part-time professionals	6	20	7.54	.006**
Full-time professionals	1	11	8.33	.001**
Speedrunners	4	4	0	1

All distributions were tested against equal distributions using the *χ*^2^ statistic. A significant *χ*^2^ test implies an unequal distribution of people between the two clusters for any given self-identified gamer type. For example, casual gamers are split 50/50 between the two clusters and thus the *χ*^2^ test is non-significant. However, full-time professionals overwhelmingly fall into the second cluster and thus the *χ*^2^ test is statistically significant. Note that the test outcome can be interpreted with more confidence if the underlying sample is larger.

* = *p* < .05.

** = *p* < .01.

*** = *p* < .001.

Based on this clustering process, the two groups, as expected, differ drastically with regard to their scale values (see Table 4).

**Table 4:** Cluster means and descriptive values for questionnaires.

	Cluster 1: tolerate	Cluster 2: troubled
Concerns		
Food supplements	11.07 (15.99)	37.02 (26.46)
Drugs (accepted)	17.24 (18.00)	54.83 (25.73)
Drugs (not accepted)	40.73 (28.74)	72.46 (21.67)
Brain stimulation	46.16 (28.40)	67.93 (20.95)
Pharmaceuticals	44.97 (29.94)	65.89 (23.78)
Fairness		
Food supplements	4.70 (0.59)	3.47 (1.12)
Drugs (accepted)	4.58 (0.63)	2.91 (1.11)
Drugs (not accepted)	4.06 (1.05)	2.27 (1.07)
Brain stimulation	3.84 (0.97)	2.73 (0.87)
Pharmaceuticals	3.62 (1.2)	2.63 (1.03)

SDs in brackets.

**Figure 3:**
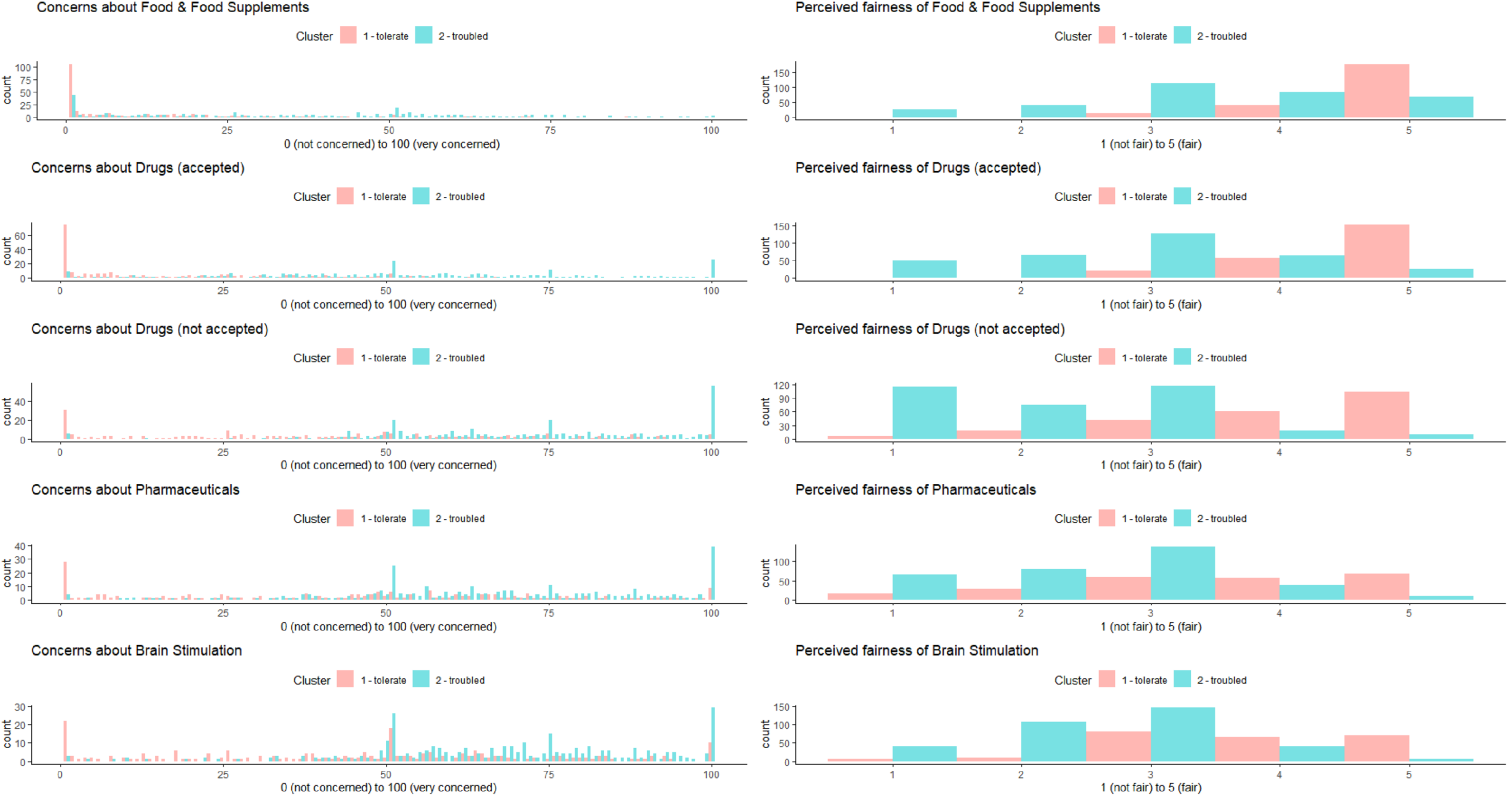
Perceived fairness of and concerns for enhancers as a function of the cluster. The y-axis indicates the number of times this value was chosen as a reply.

Figure 3 shows a distribution of values across clusters and questionnaires in a histogram.

In sum, based on these results we can conclude that there is some evidence that players differ in their perceived fairness and concerns based on their player type. Specifically, the more time an individual invests in esports and the more important competitive gaming is to them, the more likely it seems that an individual possesses concerns about the use and fairness of performance enhancers in the context of esports.

### 4.5 RQ2: how do players rationalize their opinions about fairness, ethics, and regulations of performance enhancers in esports?

We employed two lines of questioning in prompting participant perspectives. In one line of questioning, we asked participants about their opinions on performance enhancers in general; in the second, we additionally posed questions about each specific enhancer type. All participants received all questions. The replies to the enhancer-specific questions were analysed in concert with the general questions. The analysis consisted of two parts. First, the responses were coded based on whether the participants considered enhancers fair using the pre-defined codebook (all unfair, all fair, depends on the enhancer, depends on the situation, depends generally). This data was contextualized with additional information from the coding. We report this data in Section 4.5.1. Second, the data was further analyzed in thematic analysis, as described earlier and reported in Section 4.5.2.

#### 4.5.1 Frequency of fairness codes

A similar number of participants generally thought that all enhancers were fair (*n* = 25, 7%) or unfair (*n* = 24, 7%). The vast majority of respondents indicated that whether or not an enhancement is fair or unfair depends on the enhancer (*n* = 141, 41%), the situation (*n* = 93, 27%), or just in general (*n* = 37,11%). For an overview of how participants’ assessment of fairness related to additional factors, please refer to Table 5.

**Table 5:** Participants were asked to indicate in what context enhancement may be considered fair or unfair to use.

Primary classifiers	All unfair	All fair	Depends on the enhancer	Depends on the situation	Depends generally
Additional classifiers					
Legality/against rules	1	3	27	13	9
Availability	1	1	27	14	8
Treat it the same as real sports	0	1	0	1	1
Only when performance is really enhanced	0	1	53	19	19
Only when medically necessary	2	1	31	44	9
Side effects on health	0	2	17	9	11
Only in tournaments/high level play	0	0	5	9	2
Only food/accepted drugs/natural	0	0	47	7	0

The table displays the frequency of replies that fell into certain categories. Additional classifiers were used to further describe the responses; for example, a person may state it depends on the enhancer in question, and clarify in addition that they would be fine with people using pharmaceuticals only for medical reasons. Consequently, the secondary classifiers give nuance to the primary meaning of a response to the question. Note that one response may be coded with more than one additional classifier.

#### 4.5.2 Thematic analysis

Through our thematic analysis, we developed a set of four themes that highlight community discussions and perspectives on the fairness of performance enhancers in esports. Some of the themes are somewhat contradictory, as the themes highlight a diversity of opinions around the fairness of performance enhancers, as well as a tension between the impact of enhancer usage on health and competitive integrity.

##### 4.5.2.1 Regulate enhancer use to derisk esports

Many participants felt that enhancer usage created risks for esports, and that increased regulation may be a valid approach to negating some of those risks. While some participants mentioned specific areas of esports that could be better regulated, there were two broad risk categories that were identified as being primed to benefit from regulation. In particular, people view the health of competitive esports professionals as a significant area of concern that needs to be addressed. There has been growing speculation within the esports community that many professional players may be using performance-enhancing substances in order to establish a competitive advantage (6). Overall, there is an appetite among the community to ensure that the health and wellbeing of players are protected through regulatory efforts. In general, the discourse in the community focused on increased regulation to restrict potentially harmful enhancers, such as pharmaceutical drugs—especially for those without a valid prescription. There was a general belief that existing regulatory efforts in this area have not gone far enough to protect player health and players are aware that abuse by (senior) professional players has a knock-on effect on other people in the scene (e.g., “I do see young players potentially copying certain ‘habits’ of successful players which could lead to future addiction.”). Opinions around enhancer use differ substantially, but most people seem to agree that when detrimental health effects occur, it has implications on fairness. “I think enhancers are fine/fair to use in all contexts except for those wherein it is evident that the player is sacrificing an obscene amount of their health and well-being for the result.” In a similar vein, people viewed increased regulation as a means to mitigate the risk to the integrity of esports more generally. This group of participants believed that regulation could act as a mechanism to reduce enhancer use, thereby levelling the playing field among competitors (e.g., “I think they would be considered fair game, because brains are unique, and some need help to be on an even playing ground.”). There was a particular concern about making sure that everyone was competing on even footing. The majority of participants did not indicate what type of governing body ought to be involved in regulatory efforts—although amongst those that did provide input, there appeared to be a preference for regulation at the esports community level (e.g., regulation within an esports league), rather than governmental regulation. Despite this, some participants did caution against “…delegating regulatory and investigatory authority into regularly officials in a nascent field such as esports.” Nevertheless, the legality of an enhancer does not necessarily prevent its accessibility and use: as with other uncontrolled substances, enhancers may be widely available regardless of their legality. To this end, participants noted that “I somewhat see the global legal situation with drugs as being unfair for players. If e.g., player A lives in a country where psychedelic or rather holotropic drugs…are legal/decriminalized and player B lives in a country where psychedelic drugs are illegal…it is obvious that player A might have an advantage over player B.”, and, “Unfair advantage is anything that a competitor doesn't have access to that another does.”

##### 4.5.2.2 The only moral enhancement is my enhancement

A relatively large group of participants felt that enhancers have the potential to be unfair or unethical to use, although they generally considered their own use of enhancers to be fair or otherwise justified. For example, people who drink coffee will typically consider enhancers like caffeine to be fair, providing the justification that it only helps players to stay awake—while other enhancers may be unfair because they alter performance. While one interpretation may be that people simply believe that their enhancer usage wasn't that serious, this pattern of response was seen across virtually all enhancer types (e.g., for food supplements “They help, I don't see it being unfair though. I use Gorilla Mind Smooth personally”, or alcohol “Whenever I get tilted I tend to grab a bottle of alcohol and get tipsy.”). Viewing “other” enhancers as problematic suggests that esports communities will face difficulties reconciling regulation that directly impedes their own enhancer use cases. In a related vein, participants who self-disclosed drug or pharmaceutical usage often highlighted that they were justifiably “leveling the playing field” by mitigating a perceived performance deficiency. Some disclosed conditions such as ADHD, and that prescription stimulants such as Adderall were important to their day-to-day function. In cases where people disclosed having a prescription medication, they almost always advocated that prescription use ought to be exempt from any regulations in the context of esports. Participants who did not disclose that they had a prescription instead made health-related arguments, such as that if they were to cease using opiates they would not be able to play.

##### 4.5.2.3 The fairness of use depends on the enhancer and its effects

Many participants felt that the performance-enhancing effects of the enhancer should be a factor in whether or not they are considered fair or unfair. Some participants understandably felt that there is a relatively large gap in effect between a food stimulant like coffee and a pharmaceutical stimulant like Adderall, and also consider the relative availability of these substances in their consideration: “To me, it is fair as long as the stimulant is socially normalized and widely available. Caffeine, alcohol, weed, etc. Whereas, if someone is taking heroin or using electronic brain stimulation devices, that to me is suspect.” Interestingly, it is worth noting that the social normalization of enhancers varies culturally—while alcohol and marijuana are normalized to varying extents in some cultures (e.g., The Netherlands), their use may be either illegal, heavily regulated, or socially dissuaded in others (e.g., The United Arab Emirates). As such, the use of social normalization as a yardstick would also represent significant complexity in its application. In many cases, participants perceive it as appropriate to regulate enhancers (especially pharmaceutical and drug stimulants) based on their perceived enhancement effect. In terms of where to draw the line, participants also suggested that enhancers that have a proven performance benefit should be regulated, while enhancers that do not have a proven performance effect should be unregulated. As a caveat, participants with this view often expressed that the magnitude of the effect should also be a consideration, such that household enhancers like caffeine should not be regulated. Although in principle, regulating based on effect seems sensible, in reality, this is problematic as inter-individual differences in responses to enhancers are large. This kind of regulation would necessitate further research into enhancer effects. Participants often view “levelling the playing field” as a legitimate use of enhancers: “I think it is fair to use substances that aren't actually performance enhancing, or to address medical issues you have to make your performance “normal” (e.g., Ritalin for ADHD)”. This has interesting implications for prescription drug abuses, as it highlights that the community has strong values around supporting people with legitimate medical needs.

##### 4.5.2.4 Regulation is not a simple solution

Some people also felt that efforts to increase fairness through regulation would be difficult, and potentially even detrimental. At large, participants with this perspective seem to believe that non-medical use of prescription medications ought to be regulated. However, it seems unclear which enhancers should be regulated and who makes the decisions; some players even suggest a player-driven process to decide what is regulated on a game-by-game basis (e.g., “Needs to be even playing field for both sides. Could ask other side if it’s acceptable to be fair.”) Further, they see the system for determining medical necessity as flawed. In particular, there were concerns raised that players can simply “doctor shop” to obtain prescriptions. This phenomenon creates a major barrier to regulation, in that regulators either need to exclude people who have a legitimate medical need, or open the door to non-medical use of prescription medications (e.g., “When it comes to Adderall it should be unfair to use if it's not prescribed and it should be highly regulated that the people using it with a prescription are using it in a fair manner. e.g., prescription being forged, they're not supplying it to other players/teams etc.”). Participants highlighted that this is further complicated by the jurisdictional availability of pharmaceuticals in different regions. This is especially interesting in the case of online tournaments, where competitors may be competing remotely from countries with different legal frameworks. In a similar vein, people also raised concerns about triggering a “substance arms race”, in which players willing to use enhancers may attempt to game the system, and take dangerous experimental substances in order to skirt restrictions around specific pharmaceuticals. This concern appears to stem from other sports (e.g., “If you regulate amphetamines + modafinil, teams will be bribing doctors to get their players diagnosed with adhd and they will get medical prescriptions. It will lead to untestable versions being developed and teams will get barred from playing like russia at the olympics.”). Some voices in the community even argue that any regulation outlawing certain enhancers would be counterproductive (e.g., “Almost all elite athletes use performance enhancers already, even if banned. Elite athletes will do anything to win, even if it shortens their life. Instead of keeping it as an open secret, just allow everything unrestricted, so long as the athlete themselves is the one doing the competing.”) and that drug testing itself can be a problematic endeavour (e.g., “Regulation with drug testing is useless. The test will become to easy to manipulate but allowing a certain amount and limit extreme doses can give a little but not a lot.”). There was also general consensus that regulation should not attempt to interfere with casual play, and should only exist for high-level, presumably professional esports: “You can dope yourself in semi-casual ladder gaming, when only virtual numbers of rating on the line. Other than that it‘s a no go”.

## 5 Discussion

In this section, we summarize the results of our analyses and discuss the implications with regard to the extant literature, and future regulatory efforts.

In short, our results reveal that the esports community at large is more concerned with pharmaceuticals, non-invasive brain stimulation, and socially non-accepted drugs as compared to food supplements or socially accepted drugs (vice versa for the perception of fairness). Further, the community can be divided into two clusters: those troubled by enhancer usage and those tolerant of enhancer usage. Investigation into the clusters reveals that the more time an individual invests in esports and the more important competitive gaming is to them, the more likely it seems that an individual possesses concerns about the use and fairness of performance enhancers in the context of esports. Additionally, a thematic analysis revealed four discussion themes present in the esports community: that enhancers should be regulated to derisk esports; that personal enhancement usage is typically justified by the individual; that fairness is dependent on the enhancer, as well as its effects; and that regulation is complex and multi-faceted.

### 5.1 Fairness by enhancer

With regards to fairness and concerns, participants agreed that pharmaceuticals, brain stimulation, and not socially accepted drugs were similarly both highly concerning and least fair. For comparison, the concern was twice to thrice as high as compared to food supplements and socially accepted drugs. The difference was less pronounced in the fairness ratings, but even there a difference of 20% is observed.

### 5.2 Players’ thoughts about fairness and concerns

Dedication to a game seems to impact the perception of enhancers and generally speaking: more dedicated players were more concerned about enhancers and perceive them as less fair. These results support the notion that the higher the degree of importance for competitive gaming in an individual's life, the more likely they will be troubled by the possibilities of illegitimate enhancement methods. We suggest that there are multiple potential factors that may explain this distinction: first, that the more important a role competitive gaming play in an individual's life, the higher the regard they have for the sanctity of the format (that is, that these players are less likely to perceive competitive gaming as “just a game”). Secondly, these players may be more likely to expect that they may eventually compete against players with an enhanced advantage (or, conversely, be in a position in which they may feel compelled to employ enhancers). Third, said players may already have negative personal experiences with enhancers in competitive formats (regardless of disclosure). We note that many player discussions have centered around the topic of equal opportunities in a competition, so that winning is determined by skill and not affected by other factors. Thus, although the players don't use the term, justice is important (38). Community members’ voices echo the notion that distributive justice can only be achieved if transparent procedures are in place to ensure that the competition outcome is based on skill rather than for example access to resources such as performance enhancers or bribes for judges. Put differently, if access to and restrictions of performance enhancers are not equal for everybody and based on the same set of rules, any outcome of a competition can be questioned.

In the end, these results are comparable to results obtained investigating fairness perception in more traditional sports [e.g., (12, 29, 49, 56)]. Thus there seems to be a shared commitment to “fair play” emphasizing the universal values of sportsmanship, discipline, and healthy competition, transcending the boundaries between virtual and physical sports. This not only upholds the integrity of competition but also promotes a sense of equity among athletes and fans alike. The “Spirit of Sport” can be preserved by making sure the perception of fairness is held high and that the will to succeed does not lead to circumvention of rules and the use of illicit performance enhancers (57, 58).

### 5.3 Regulatory implications

The results make it clear the regulators and other stakeholders in the esports industry need to consider how to go forward. One such issue that needs to be addressed in the future is the use or abuse of pharmaceuticals. If regulators decided to ban the use of pharmaceuticals that are not medically needed, a well-known problem emerges. Although in theory, the ‘therapeutic use exemption’ should only affect people that actually need a certain pharmaceutical to overcome a limitation, in reality, people will find a way to gain access to that exemption via illegitimate means (e.g., bribery, forgery) or circumvent the drug-screening process (12). Thus a harder regulatory stance may be taken, but that may also lead to wrongful denial of medication. There have been cases where athletes even removed themselves from competition after a therapeutic use exemption was denied (28). Thus anything that would be considered typically as doping (such as abusing pharmaceuticals beyond the therapeutic use exemption) is, ethically speaking, discouraged as it undermines the “spirit of the sport” as the WADA puts it. However, there is also a problem with supposedly ethical performance enhancement that seeks only to optimize performance through transparent and acceptable means, contributing positively to the perceived fairness of sports. Where do the boundaries between doping and ethical performance enhancement lie? Who makes those rules? Is it ethical when only a portion of the athletes have access to legitimate performance enhancement due to for example high-costs? Striking a balance between pushing the boundaries of human potential and maintaining the integrity of fair competition—also in the eyes of the outside observer—is tremendously difficult but crucial in navigating the ethical landscape of sports performance.

Further, regulators should remain cognizant of positioning esports as an inclusive and equitable space. While esports does currently pose significant barriers to participation from marginalised groups [e.g., the presence of discriminatory behaviours and expectations directed at women; see (59–61)], the medium in which esports occurs erases many of the physiological disparities present in physical sports. As such, regulators should seek to maintain the more equitable advantages of this novel sporting context—and be aware of the potential of regulations and drug testing procedures to discriminate against groups of people. Regulators may turn to other “mind sports”, such as chess, for guidance on the matter. The International Chess Federation (FIDE) abides by the general WADA rules and stresses the importance of certain potential stimulants for chess. Specifically, FIDE prohibits the use of stimulants such as pseudoephedrine, amphetamines, ephedrine, and modafinil. Notably, while substances such as caffeine and codeine are not strictly prohibited, they are monitored. However, given the history of doping scandals in WADA regulated sports it seems prudent to assume that some chess players circumvent regulations. Nevertheless, chess may be similar enough to look for inspiration on anti-doping regulations. Although chess may be used as inspiration for regulators, there are distinct differences between chess and esports. Unlike chess, esports places a considerable emphasis on motor skills, expanding the scope of necessary regulations. The dexterity involved in esports present distinctive challenges that go beyond the cognitive demands found in chess. Specifically, in chess, the FIDE prohibits stimulants primarily targeting brain activity. Although similar substances can be problematic in esports as well, the regulatory landscape in esports needs to consider a broader spectrum of performance enhancers, including those targeting direct muscle activity and analgesics to numb pain from carpal tunnel syndrome.

In general, any regulatory effort needs to consider how athletes are treated and what image is projected onto them: are athletes presumed to be innocent or guilty? If an athlete is presumed to be guilty from the beginning, the athlete may decide to conform to those assumptions and break the rules. One way to potentially reduce cheating behavior is to change the way unfair behavior is discussed and how fairness is promoted. For example, Bryan and colleagues (62) showed that people were less likely to cheat when the framing of pro-fairness slogans implies that cheating is diagnostic of an undesirable identity. However, Bryan et al. also point out there are issues with using this approach. First, false positives when detecting cheaters may result in individuals falsely integrating cheating behavior into their identity; second, this approach relies on the fact that the individual sees themselves as a good person, and does not want to cheat. As such, a person that wants to play unfairly will not be affected. A further problem is the perceived fairness of the regulation itself as there is for example a danger of false positives or procedurally unfair procedures leading to a disqualification of athletes (63, 64). For example, recently Sun Yang, a Chinese Olympic-Gold-Medalist in swimming, was banned for several years because it was deemed proven he interfered with a blood sample (65). However, observers and researchers have argued that this trials may not have been fair because of inadequate translation services and the athlete had his right to be hear infringed. The problems with tests themselves are further exacerbated when considering trans athletes and steroid users wanting to return after a suspension (66, 67). Although sex-specific doping or steroids in esports seem to be to a lesser degree concerning in esports, the core issue of subjectively unfair regulations remains.

An additional challenge arises based on the structure of the esports system. Esports is driven by companies with the goal of maximizing profits. Consequently, the health of the athletes as well as the propagation of fair competitions may only be a means to increase profit and not inherently valuable to a company. For example, the gaming and esports market is heavily targeted by energy drink companies, such as “Red Bull”. Red Bull sponsors esports teams around the globe and even finances competitions. Although in isolation this may not be problematic, the heightened consumption of energy drinks may not only be problematic for health reasons but undermine anti-doping policies in general (68). Similarly, nootropic manufacturers (e.g., HOLY, LevlUp) or brain stimulation companies (e.g., halo, omnipemf) may soon start sponsoring esports teams to increase their public exposure. All of these potential enhancers have an inherent health risk associated with them, with no guarantee to actually enhance performance in an individual. So the question needs to be asked whether or not a ‘potential’ enhancer should be regulated or not, and what regulations should apply for “potentially” unhealthy substances. What can be possible concerning is the match between energy drinks as a product and esports, resulting a powerful marketing force (69). Undoubtably high-glucose energy drinks are unhealthy, especially if consumed in larger doses, and they are already marketed towards younger individuals, which overlap with the audience consuming esports content (70). Given that esports itself as well as its regulation is de-centralized and driven by commercial interests of the companies owning the game being played, there is a conflict of interests that may negatively affect the health of both athletes and consumers of the sport (71, 72).

Further, our results revealed a strong community sentiment towards only regulating enhancers that have been proven to increase performance. However, this implies that actual research exists and that the results are conclusive. While for some enhancers, such as cocaine, such research may be unethical, other performance enhancers only improve performance in some individuals. For example, non-invasive brain stimulation via transcranial direct current simulation results in large inter-individual differences (73, 74). As such: while the community sentiment on this subject is largely cohesive (that is, apply regulatory restrictions only to enhancers with a proven enhancement gain), the path towards collecting evidence to support these regulations is fraught, poses ethical concerns, and requires a large body of work. Consequently, regulation reliant on this motivation may currently be hamstrung by a lack of empirical guidance.

Another side-effect of being a company-driven sport is that no overarching governing agency exists that has the power to enforce rules. Whereas in Olympic sports the World Anti-Doping Agency (WADA) possesses significant authority, and most national sports bodies operate within its frameworks, there is no equivalent in the esports scene. The Esports Integrity Coalition (ESIC) is one organization that aims to become a leader in that regard, but other organizations such as the World Esports Association (WESA), which was established by a tournament organizer, the Electronic Sports League (ESL), and the International eSports Federation (IeSF) claim overlapping responsibilities. Currently, each tournament organizer can effectively publish their own set of rules. As a consequence rules across and even within sports (games) may not be consistent, procedures not transparent, and regulations unequally enforced. For example, while League of Legends developer Riot Games holds its own tournaments, the premier organizer of Starcraft tournaments is ESL and not the game developer itself. While within a certain community a specific organization can be viewed as the legitimate governing body, no organization can currently claim legitimacy across esports as a whole. An organization claiming to legitimately govern the whole of esports would need to provide a regulatory ruleset that is perceived to be fair by the majority of the community, but especially the competitors.

### 5.4 Limitations and future research

While this work makes important contributions to our understanding of enhancer perceptions, the study has several limitations to be mindful of. First, the gender distribution of the sample is skewed towards participants that self-identified as men. This may be an artefact of the community sampling method used. While there is good evidence to support that women comprise approximately half of all gamers, women have been disproportionately underrepresented at professional esport events. Gender discrimination, harassment, and negative stereotype threat are believed to contribute to the lack of women in professional esport contexts (75–77). Future research and governing efforts should aim to further investigate women and non-binary perspectives on esports and foster a welcoming environment for all players. Second, the enhancer categories that participants were presented with could have been structured differently; specifically, the categories could have been more granular. For the purposes of simplicity and keeping the survey short, several broader categories of enhancers were constructed. However, there are many pharmaceuticals or drugs available that may impact the individual in different ways (e.g., stimulants vs. depressants vs. psychedelics). Splitting enhancers into a greater number of categories would have inflated the time to complete the survey drastically. Our approach sought to balance data quality by maintaining participant attention, while offering broader categories. The categorization of performance enhancers utilized in this research is notably broad and lacks specificity concerning sport-related enhancements, which is particularly pertinent in the context of esports. Additionally, the chosen categories may incorporate a degree of bias, as terms like “drugs” carry normative implications. Nevertheless, future studies that focus on pharmaceuticals and drugs only should use a more fine-grained approach. Third, different self- identified gamer types are represented in the sample with varying frequencies. We tried to recruit as many professional players as possible by recruiting from subreddits dedicated to esports or certain esports teams. We were successful in recruiting a good number of professional players (112 individuals, 19.79% of the sample, reported to play at least at an amateur level). Fourth, there are known limitations with self-report measures and questionnaires, such as social desirability biases or tendencies towards the mean. Although we cannot fully exclude these issues, distribution analysis shows reasonable standard deviations (see Tables above) and distribution parameters. Specifically, the interquartile range for enhancer concern (rated from 1 to 100) was between 32 and 48 depending on the enhancer and between 1.5 and 2.2 for fairness perception (rated from 1 to 5). The range of values incorporated both extreme values for each enhancer for both concern and fairness scores. Future research could make use of implicit measures that aim to circumvent potentially problematic self-report issues (78). Fifth, we only added a scale measuring distributive justice, even though other facets are also important. Distributive justice evaluations focus on the outcome of a process, rather than the evaluation of the process itself. Evaluating whether or not a person or team subjectively deserves to win is possible for all participants. However, evaluating the process, the interactions with officials or the flow of information may not be possible for all study participants. Studying other aspects of justice required either the creation and evaluation of vignettes or the focus on people with insider information (e.g., esports professionals). Sixth, justice and injustice as well as their consequences, are potentially difficult to study because experimental manipulations can only be done on a small scale, otherwise, they would be unethical. Thus researchers rely on the creation of vignettes, questionnaires, interviews, and the post-hoc evaluation of certain events. Nevertheless, these subjective perceptions are real and shape behavior, even if they are not necessarily routed in facts.

## 6 Conclusion

This work investigates the perception of performance enhancer usage in esports contexts. Analysing the data both quantitatively and qualitatively, we investigated fairness and concerns surrounding performance enhancer usage as well as the regulatory implications. Results show that the competitive gaming community at large differentiates between potential performance enhancers and is most concerned about “hard” drugs, pharmaceuticals as well as brain stimulation interventions (vice versa for fairness judgements). Socially accepted drugs and food or food supplements seem to be more accepted. Further, people that are more invested in the competitive gaming scene tend to be more skeptical of performance enhancers and tend to have bigger concerns. Understanding how the competitive gaming community thinks about enhancers can inform future regulations. The fragmented regulatory landscape in esports may lead to a different perception of tournament winners based on which governing body supervised the competition. The perception of fairness of a competition is key to that competition and its outcomes being perceived as legitimate. If an institution (e.g., a tournament organizer) can guarantee a competition that is largely perceived as fair, the organizer as well as the outcome will be more likely to be perceived as legitimate. We suggest that regulators involve researchers as well as their playerbase (e.g., in the form of a community or an esports athlete council) in an transparent decision-making process when it comes to tournament rules and regulations. In turn, a transparent decision-making process may result in a higher acceptance and perceived legitimacy of a decision. The present results further highlight that esports and traditional sports are not that different. In fact, the present finding resonate with traditional sports literature, which may not be surprising given the more recent professionalization of esports and it being picked up by established sports teams (such as Paris Saint-Germain or Schalke04).

## Data Availability

The raw data supporting the conclusions of this article will be made available by the authors, without undue reservation.

## Footnotes

^1^Note that we did not use legality as a classification system. This is because the rules and regulations surrounding different potential enhancers, drugs, and pharmaceuticals can differ drastically between countries. We acknowledge that different categorization systems may influence survey outcomes.

^2^Please note that we collected additional data in this questionnaire. However, these data are beyond the scope of the present manuscript and are reported elsewhere. For details, see ([25])

^3^Note that the drastically unequal groups sizes in self-identified gamer categories preclude us from conducting standard pairwise comparisons.
