# Supplementary material for: Fighting fair: community perspectives on the fairness of performance enhancement in esports
Source: Front Sports Act Living. 2024 Mar 12;6:1330755. doi: 10.3389/fspor.2024.1330755 (PMC10963441; doi:10.3389/fspor.2024.1330755)

**Supplemental Material**

**Subreddits where the survey was advertised:**

https://www.reddit.com/r/Nootropics/

https://www.reddit.com/r/Biohackers/

https://www.reddit.com/r/steroids/

https://www.reddit.com/r/opiates/

https://www.reddit.com/r/speed/

https://www.reddit.com/r/StackAdvice/

https://www.reddit.com/r/Competitiveoverwatch/

https://www.reddit.com/r/R6ProLeague/

https://www.reddit.com/r/CompetitiveForHonor/

https://www.reddit.com/r/CompetitiveMinecraft/

https://www.reddit.com/r/CoDCompetitive/

https://www.reddit.com/r/FortniteCompetitive/

https://www.reddit.com/r/starcraft/

https://www.reddit.com/r/QuakeChampions/

https://www.reddit.com/r/Smite/

https://www.reddit.com/r/truegaming/

https://www.reddit.com/r/hearthstone/

https://www.reddit.com/r/DotA2/

https://www.reddit.com/r/MagicArena/

https://www.reddit.com/r/TrackMania/

https://www.reddit.com/r/vainglorygame/

https://www.reddit.com/r/teamliquid/

https://www.reddit.com/r/pcmasterrace/

https://www.reddit.com/r/titanfall/

https://www.reddit.com/r/OverwatchUniversity/

https://www.reddit.com/r/haloinfinite/

https://www.reddit.com/r/truetf2

Other subreddits were contacted but either did not approve of survey posts or did not get back to the authors.

**Cluster analysis figures**

Scree Plot


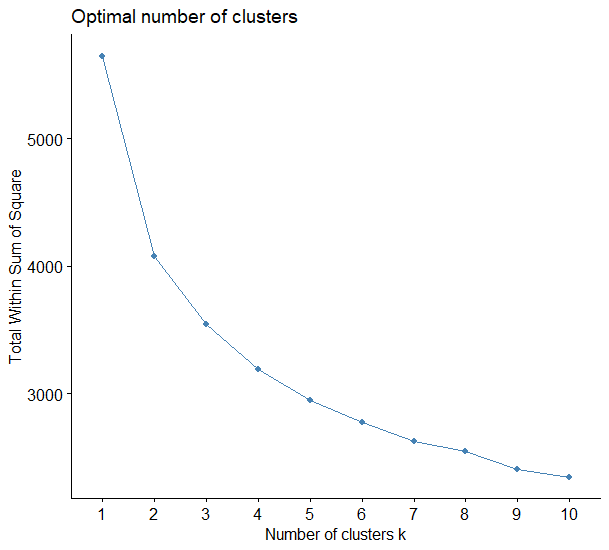


Silhouette Plot


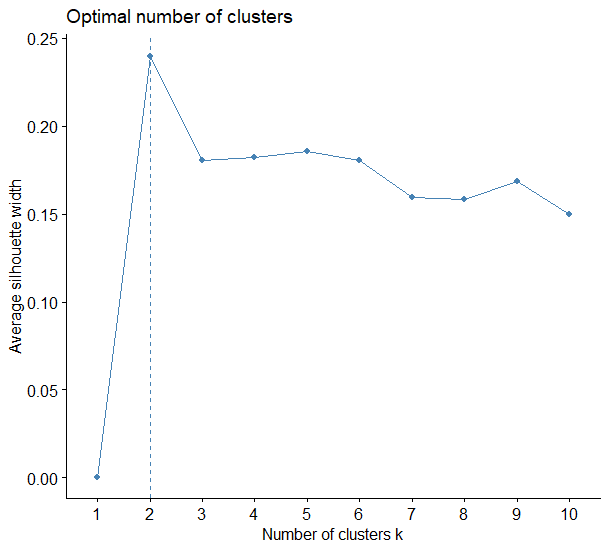


Best point separation plots


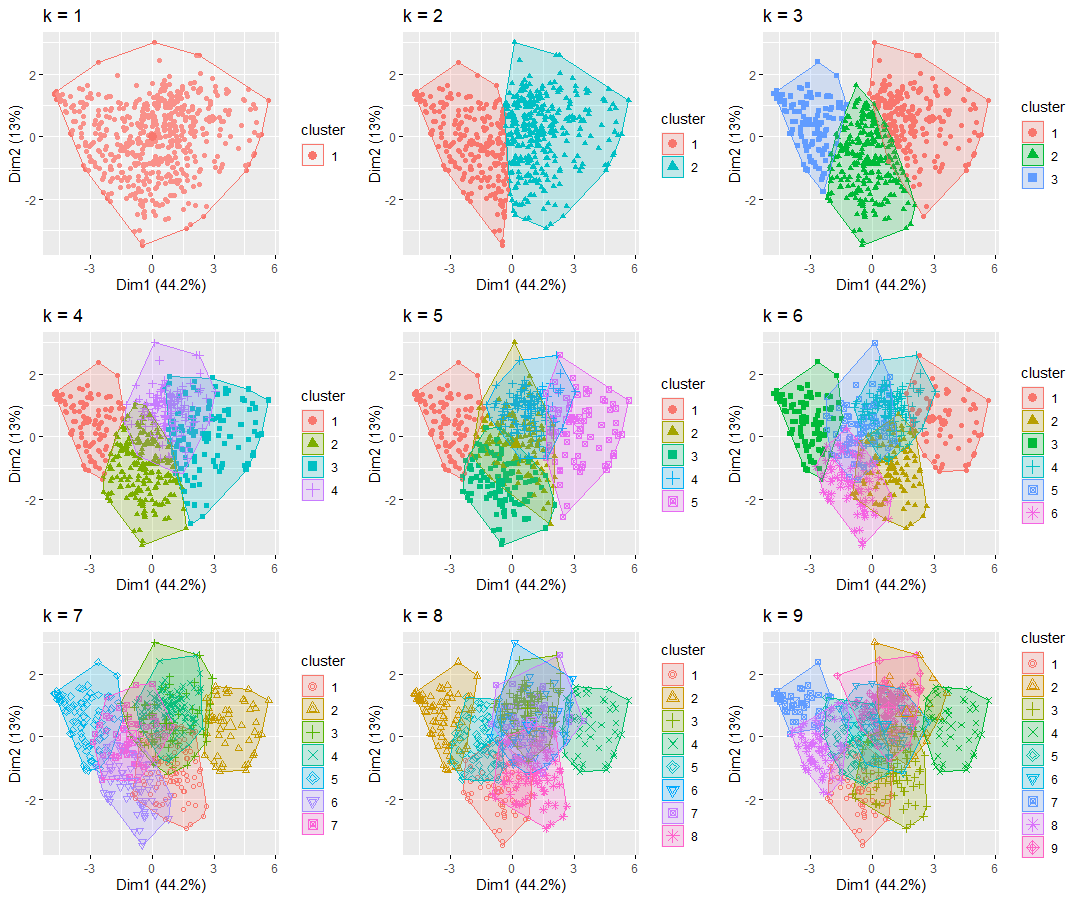

Supplement: Supplementary file 1 [file Datasheet1.docx]
